# The complete mitochondrial genome of red-throated flycatcher *Ficedula albicilla* (Passeriformes: Ficedula)

**DOI:** 10.1080/23802359.2019.1673682

**Published:** 2019-10-03

**Authors:** Xiao-Ran Zhang, Cai-Hong Lu

**Affiliations:** a Jiangsu Provincial Nanliuji Township People's Government, Jiangsu, China;; b College of Biology and the Environment, Nanjing Forestry University, Nanjing, China

**Keywords:** *Ficedula albicilla*, mitochondrial genome, red-throated flycatcher, Ficedula

## Abstract

We report the mitochondrial genome of *Ficedula albicilla*. The overall base composition of *F. albicilla* mitogenome is 29.49%A, 15.06%G, 32.98%C, and 22.47%T, with an A + T content of 51.96%. The total length of the sequence is 16,791 bp (13 protein-coding genes, 22 transfer RNA genes, 2 ribosomal RNA genes, and 1 control regions). Phylogenetic analysis was performed based on the concatenated nucleotide sequences of cytochrome c oxidase subunit I and cytochrome b using the neighbor-joining method and the Kimura 2-parameter model in MEGA 7.0 with 1000 bootstrap replicates.

Red-throated flycatcher (*Ficedula albicilla*) is a kind of small bird usually seen in Origin, about 11–13 cm in length. *Ficedula albicilla* belongs to the scientific class chordate phyla, Aves, Passeriformes, Muscicapidae, Ficedula. This species distributes widely in Eurasia and appears in East Asia during the migratory season (Ergen and BarIş 2016). The previous results suggest that *Ficedula parva albicilla* and *F. p. parva* should be two species, However, the conventional view among zootaxonomist was that they were a subspecies of *Ficedula* (Li and Zhang 2004).

This study reports, for the first time, the complete mitochondrial genome (mtDNA) sequence of *F. albicilla*. Samples were collected at the Hongze Lake Wetlands National Nature Reserve (33°14′N, 120°19′E) in the east of China in October 2018 and after sampling, the specimens (NJFU-2018082) were stored in the animal specimens museum of Nanjing Forestry University.

The complete mitochondrial genome of *F. albicilla* is a typical circular DNA molecule with 16,791 bp in size (GenBank accession: MN125374). The nucleotide composition is significantly biased (A, G, C, and T was 29.49%, 15.06%, 32.98%, and 22.47%, respectively), with A + T contents of 51.96%. There are 22 transfer RNA genes (tRNA), 13 protein-coding genes (PCGs), 2 ribosomal RNA genes (rRNA), and 1 control region in the mitochondrial genome arrangement (Liu et al. 2019; Sun et al. 2019). The structure of the mitogenome is a typical vertebrate mitochondrial gene arrangement.

In this study, phylogenetic analysis of the *F. albicilla* and 15 other birds was carried out based on the concatenated nucleotide sequences of cytochrome c oxidase subunit I (COI) and cytochrome b (Cyt b) using the neighbour-joining method and the Kimura 2-parameter model in MEGA 7.0 with 1000 bootstrap replicates (Kumar et al. 2016).

The mitogenome of *F. albicilla* was genetically closest to that of *Cyanoptila cyanomelana* (Figure 1). The genome information obtained here could contribute to the conservation and utilization of Ficedula and Muscicapa (Sun et al. 2016).

**Figure 1.**
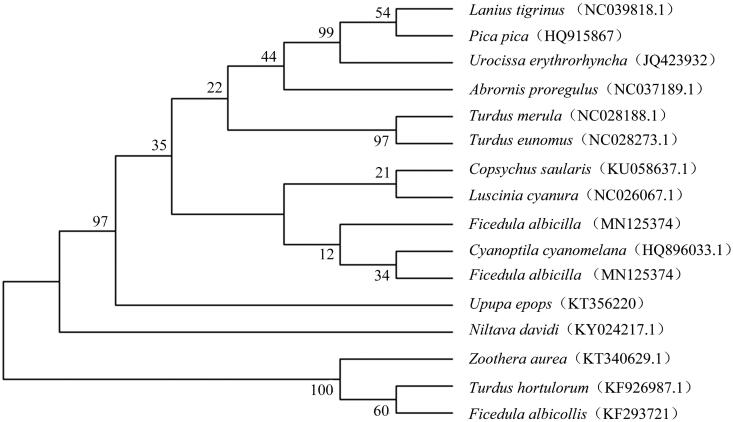
Neighbor-joining phylogenetic tree based on the concatenated nucleotide sequences of cytochrome c oxidase subunit I and cytochrome of *F. albicilla* and 15 other birds using MEGA 7.0.
